# Beyond the “Dominant” and “Recessive” Patterns of Inheritance

**DOI:** 10.3390/ijms252413377

**Published:** 2024-12-13

**Authors:** Anthoula Chatzikyriakidou

**Affiliations:** 1 Laboratory of Medical Biology—Genetics, Faculty of Medicine, School of Health Sciences, Aristotle University, 54124 Thessaloniki, Greece; chatzikyra@auth.gr; Tel.: +30-2310999013; 2 Genetics Unit, “Papageorgiou” General Hospital of Thessaloniki, Faculty of Medicine, School of Health Sciences, Aristotle University, 54124 Thessaloniki, Greece

**Keywords:** allele specific expression, allelic imbalance, single nucleotide polymorphism, genetic variant, epialleles, methylation, CpG islands, variant classification, genetic heterogeneity, Mendelian inheritance, non-Mendelian inheritance

## Abstract

This study aimed to investigate whether genes with different modes of inheritance differ in the presence of promoter-enriched CGI loci. For each autosomal chromosome, the author searched for variations in the total number of diseases’ phenotypes with autosomal dominant (AD) and recessive (AR) inheritance for a list of promoter-poor CGI (CGI−) and promoter-enriched CGI (CGI+) genes using the OMIM database. Then, the CGI− and CGI+ genes displaying random allelic or bi-allelic expression were examined. The author evaluated whether there was a distinct distribution of AD and AR diseases in the groups of chromosomes based on their SNP hotspot density. The same analysis was conducted for the X chromosome. The SPSS statistical package was utilized. The distribution of AD and AR diseases between CGI− and CGI+ bi-allelic genes significantly differed in autosomal chromosomes 6 and 17, which show intermediate SNP hotspot density. Additionally, a statistically significant difference was observed in AD and AR diseases in the remaining autosomal chromosomes with low SNP hotspots between their randomly allelic expressed CGI− and CGI+ genes. Specifically, AD diseases were related to CGI− genes, while AR diseases were associated with CGI+ genes. In the X chromosome, X-linked dominant (XLD) diseases were mainly found in CGI+ genes, and X-linked recessive (XLR) diseases were found in CGI− genes, regardless of the X-inactivation process. It is essential to study inheritance and classify genetic variants in a more stochastic way than the terms “Dominant” and “Recessive,” and their derivatives, such as “Codominant” and “Incomplete Dominant,” are applied in Mendelian and non-Mendelian inheritance. This concept may further explain the “Reduced Penetrance” and “Variable Expressivity” in certain human diseases. All the above suggests a need to reassess how genetic and epigenetic data are studied and utilized for genetic counseling or precision medicine.

## 1. Introduction

Random allelic imbalance refers to the unequal expression of alleles of the same gene in a single cell. Recently, the author reported that single nucleotide polymorphisms (SNPs), now known as single nucleotide variants (SNVs), appear to function as stochastic oscillators in the inheritance of human diseases [1,2]. Genes associated with dominantly inherited diseases are typically located in low SNP hotspot regions of human chromosomes, meaning chromosomal loci with low SNP densities, while genes linked to recessively inherited diseases are often found in dense SNP hotspot regions. Therefore, the haplotype for a number of SNPs, which is a group of alleles inherited together within an organism, is expected to impact allele-specific expression and influence Mendelian or non-Mendelian inheritance principles [1]. This could also lead to misclassifications of genetic variants associated with specific phenotypes [2].

SNPs and other genetic traits can result in allele-specific expression (ASE) between the two alleles of a gene, causing one allele to be preferentially expressed over the other despite humans being diploid. Studies suggest that 30% to 56% of genes exhibit allelic imbalance, indicating that allele-specific effects have widespread impacts on gene regulation [3]. Depending on the gene’s function, these expression imbalances can lead to phenotypic variations with functional consequences. Sequence-dependent traits can influence allele-specific expression through *cis*-acting, *trans*-acting, or *distant*-acting genetic variants on gene transcription and post-transcriptional processes [4]. Additionally, parent-of-origin-dependent ASE can occur when alleles are differentially expressed based on the contributing parent and X-linked genes may exhibit mono-allelic expression due to random X chromosome inactivation in female cells [3,4,5]. Epigenetic differences resulting from genetic variations between alleles can also lead to ASE through a process known as sequence-dependent allele-specific methylation or histone acetylation impacting non-imprinted, autosomal genes in a tissue- and individual-specific manner [4,6]. 

The human genome contains approximately 30,000 CpG islands (CGIs), which are long stretches (0.5–2 kb) of DNA with elevated levels of CpG dinucleotides [7]. CGIs typically overlap with promoters’ transcriptional start sites (TSSs) for a significant portion (60–70%) of human genes and are stably hypomethylated in various cell types [8]. DNA methylation at CpGs plays a crucial role in gene expression regulation, with limited methylation at CpGs clustered in promoters (CGI+) and more methylation at CpGs not clustered in promoters (CGI−). Intragenic and intergenic CGIs are often methylated and seem to overlap with cryptic promoters and enhancers that function in a tissue- and cell-type-specific manner [9]. 

DNA methylation is a major epigenetic modification of the genome, essential for cellular reprogramming, tissue differentiation, and normal development [10]. Therefore, alterations in epigenetic patterns have been linked to many human diseases [11]. Approximately 5% of CpG sites show a significant imbalance in DNA methylation [4]. Epigenetic differences between alleles can lead to ASE in non-imprinted autosomal genes and X-linked genes [4,5]. Given that dominant diseases (AD) are mainly found in low SNP chromosomal regions and recessive diseases (AR) in dense SNP chromosomal regions [1,2], the question arises of whether SNP density affects the possible methylation status of genes and their inheritance pattern. Therefore, the author has studied if genes with different patterns of inheritance across each chromosome have differences in the presence of their promoter CGI+ and CGI− loci. The same was tested in relation to the SNP density of chromosomes, grouping their genes as CGI+ and CGI−. 

## 2. Results

For autosomes, the distribution of AD and AR diseases between CGI− and CGI+ genes was significantly different on chromosome 6 (*p*-value = 0.019; OR: 1.864, 95%CI: 1.105–3.146) and chromosome 17 (*p*-value = 0.028; OR: 1.632, 95%CI: 1.055–2.525) (Table 1). The odds ratio reveals that AD diseases in both chromosomes are mainly found in CGI− genes, which are susceptible to methylation, while AR diseases in both chromosomes are mainly found in CGI+ genes, which are not susceptible to methylation. These chromosomes, which have an intermediate level of SNP hotspot frequency [1,2], may allow another mechanism, methylation, to cause the observed difference in expression of dominant and recessive genetic variants between their CGI− and CGI+ genes. These genes are known to differ in their susceptibility to methylation [8], ultimately leading to the reported allele-specific expression of chromosomes 6 and 17 [12].

The next analysis tested a difference in AD and AR diseases’ distribution between CGI− and CGI+ genes related to random allelic or bi-allelic expression in the group of chromosomes showing low SNP hotspots, high SNP hotspots, or in chromosomes 6 and 17. The reasons for this grouping are explained in the methodology. For chromosomes 6 and 17, a statistically significant difference was observed for AD and AR disease distribution of CGI− and CGI+ genes in case they are bi-allelic expressed (*p*-value = 0.010), which may explain the correlation in Table 1. The odds ratio reveals that AD diseases of their bi-allelic expressed genes are mainly found in CGI− genes, which are susceptible to methylation, while AR diseases of their bi-allelic expressed genes are mainly found in CGI+ genes, which are not susceptible to methylation (OR: 1.935, 95%CI: 1.169–3.202) (Table 2).

Regarding the remaining studied autosomal chromosomes, a statistically significant difference was observed in AD and AR diseases between their CGI− and CGI+ random allelic imbalanced expressed genes in the group of chromosomes showing low SNP hotspots (chromosomes: 13, 1, 14, 21, 18, 2, 20, 5, 12, and 15) (*p*-value = 0.0123). The odds ratio reveals that AD diseases of genes related to random allelic expression in the low SNP hotspot chromosomes are common in CGI− genes, meaning in genes susceptible to methylation (OR: 1.693, 95%CI: 1.121–2.557) (Table 2). The reverse correlation exists for AR diseases of genes related to random allelic expression in the low SNP hotspot chromosomes, which are common in CGI+ genes; in other words, in genes not susceptible to methylation (OR: 1.693, 95%CI: 1.121 to 2.557). Additionally, for the genes of these autosomal chromosomes related now to bi-allelic expression, no statistically significant difference was observed in AD and AR diseases’ distribution between their CGI− and CGI+ genes in the group of chromosomes showing low SNP hotspots (chromosomes: 13, 1, 14, 21, 18, 2, 20, 5, 12, and 15) or in the group of chromosomes showing high SNP hotspots (chromosomes: 10, 3, 11, 22, 4, 7, 9, 19, 8, and 16) (Table 2).

Finally, concerning the X chromosome, a statistically significant difference was observed in the distribution of XLD and XLR diseases between its CGI− and CGI+ genes (*p*-value = 0.028; OR: 0.491, 95%CI: 0.259–0.928) (Table 1). Reversing the odds ratio value to present the positive association (OR: 2.038, 95%CI: 1.078–3.854), XLD diseases are mainly found in CGI+ genes, which are not prone to methylation, and XLR diseases are mainly found in CGI− genes, which are prone to methylation. Furthermore, when the analysis was performed for mono-allelic or bi-allelic expression of the tested X-linked genes, no statistically significant difference was revealed in XLD and XLR diseases’ distribution: (i) between CGI− genes that escape from X-inactivation vs. CGI− genes that are processed to X-inactivation, (ii) between CGI+ genes that escape from X-inactivation vs. CGI+ genes that are processed to X-inactivation, (iii), between CGI− vs. CGI+ genes that both are processed to X-inactivation (mono-allelic expression), and (iv) between CGI− vs. CGI+ genes that both escape from X-inactivation (bi-allelic expression) (Table 3).

**Figure 1:**
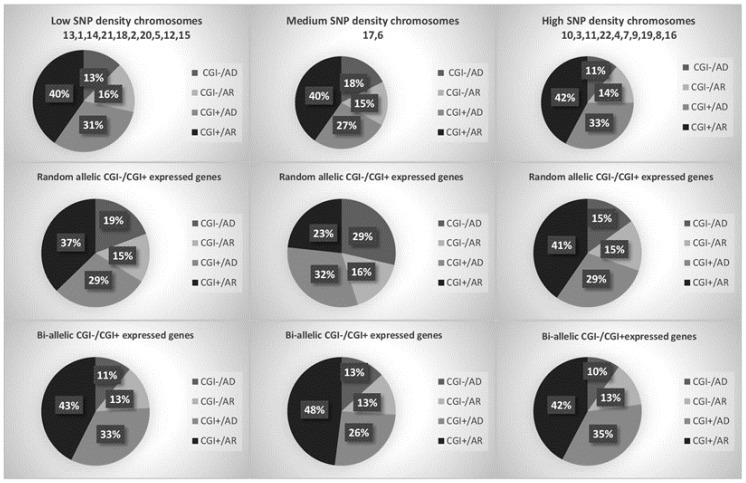
Autosomal chromosomes have been grouped based on their SNP hotspot density to display the percentage of their: (i) CGI− (promoter-poor CpG islands; CGI) genes and CGI+ (promoter-enriched CGI) genes that follow either the autosomal dominant (AD) or recessive (AR) inheritance pattern and (ii) CGI− and CGI+ mono-allelic or bi-allelic expressed genes with AD or AR inheritance pattern.

The Supplementary File contains 12 sheets from which AD, AR, XLD, and XLR diseases were retrieved for each aforementioned analysis of Table 1, Table 2 and Table 3. In Figure 1, chromosomes have been grouped by their SNP hotspot density to show how their CGI− and CGI+ genes behave in terms of inheritance patterns generally and when these genes are mono-allelic or bi-allelic expressed. 

## 3. Discussion

Initially, the present study revealed that the distribution of AD and AR diseases between CGI− and CGI+ genes was significantly different only for autosomal chromosomes 6 and 17; AD disorders were associated with CGI− genes susceptible to methylation, while AR disorders were associated with CGI+ genes not susceptible to methylation. Chromosomes 6 and 17 are in the middle of chromosome classification regarding SNP hotspot regions’ density, which cannot contribute to a specific allele expression [1,2]. However, these two chromosomes have been reported to show frequent allelic imbalance [12]. This suggests that these chromosomes with an intermediate level of SNP hotspot frequency may allow another mechanism of CGI− sequences and their positive correlation with methylation to influence the distribution of AD and AR diseases between their CGI− and CGI+ genes. Combining the results of two datasets [13,14] to focus on CGI− and CGI+ genes of chromosomes 6 and 17, which show random allelic imbalance or bi-allelic expression, it was found that AD diseases of bi-allelic expressed genes are mainly found in CGI− genes, susceptible to methylation, while AR diseases of bi-allelic expressed genes are mainly found in CGI+ genes, which are not susceptible to methylation. 

Chromosomes 6 and 17 were not grouped with other autosomes, which prevented their positive association of AD and AR diseases with CGIs from being included in the remaining autosomal grouping. For the remaining autosomes, the analysis combined the results of the two datasets [12,13] and focused on CGI− and CGI+ genes according to their random allelic imbalance or bi-allelic expression. This analysis showed a difference in AD and AR inheritance patterns only between CGI− and CGI+ genes located in low SNP hotspot chromosomes below the cutoff of chromosomes 6 and 17 SNP density. This difference was observed in chromosomes 13, 1, 14, 21, 18, 2, 20, 5, 12, and 15. 

In contrast, chromosomes with high SNP hotspot regions, above the cutoff of chromosomes’ 6 and 17 SNP density, such as 10, 3, 11, 22, 4, 7, 9, 19, 8, and 16, did not show a difference in AD and AR inheritance pattern between CGI− and CGI+ genes. Due to the presence of high SNP hotspot regions in these chromosomes, AD diseases are not common, and AR diseases mainly exist [1,2]. Therefore, the methylation status of promoter CGI loci cannot affect the mode of allele expression as it is already determined by their dense SNPs through other mechanisms [1,2,4]. On the contrary, in chromosomes with low SNP hotspot regions (13, 1, 14, 21, 18, 2, 20, 5, 12, and 15), the different distribution of AD and AR inheritance patterns of genes with random allelic imbalanced expression could be attributed to the promoter CGI loci. Genes with CGI+ are not susceptible to methylation [8,9,15] and are mainly related to AR diseases, where both alleles carrying deleterious variants are expressed and lead to disease. Additionally, for chromosomes with low SNP hotspot regions, the AD genes showing random allelic imbalanced expression were revealed to lack CGIs (CGI−) and are susceptible to methylation [8,9,15], causing probable allele-specific expression. Therefore, for CGI− genes, methylation may contribute to allelic imbalanced expression in favor of a deleterious variant, leading to disease manifestation with a dominant pattern in the low SNP hotspot regions.

The statement above agrees with the results of a previous study that suggested epigenetic modifications should be considered along with other potential factors, such as balancing selection, which can increase allele frequencies beyond what is expected from mutation-selection balance [16]. The relationships between genotypes and phenotypes in classical models of genetic inheritance are well understood. Previous research has shown that SNPs’ haplotypes are likely to impact allele-specific expression and influence Mendelian or non-Mendelian inheritance principles [1,2]. In-*cis* or in-*trans* variants may lead to an allelic imbalance in favor of the “Dominant” gene mutations, especially in chromosome regions with fewer SNP hotspots [1,2]. Additionally, Webster and Phillips have highlighted the role of epigenetic modifications in maintaining epialleles, which are alleles with different chromatin states due to methylation and can be inherited mitotically and/or meiotically. This maintenance can sometimes sustain deleterious alleles that would otherwise remain hidden [16]. Studies have also shown a reverse correlation between the expression of transcription factors and DNA methylation at their binding sites in various cell types [17]. Since SNPs can influence the binding levels of DNA transcription factors and subsequently affect the methylation status of a site [18], particularly in low SNP hotspot regions, passive DNA methylation is expected to remain stable. This stability may alternatively explain the expression of deleterious dominant genetic variants in low SNP hotspot regions, as discussed in the context of epialleles’ maintenance [16,18]. The present study further supports the correlation of AD disorders with low SNP hotspot regions [1], adding that AD diseases are mainly related to CGI− genes, which may be more susceptible to specific methylation patterns through epialleles. 

It is worth mentioning that gene promoters are evolutionarily conserved [19,20] even though methylated CpGs are the most rapidly mutating dinucleotides in the genome [21]. Therefore, epigenetic alterations, known as epimutations, can lead to “Dominant” variant expression through allelic imbalanced expression in these chromosome regions with low SNP variance [16]. Consequently, the “Dominant” and “Recessive” patterns of inheritance pinpoint specific traits of our genome. This should be taken into consideration to unveil elusive heritability factors and to overcome matters of genetic variants’ misclassifications [2]. Long-read sequencing promises significant improvement over short-read sequencing for accurate long-range haplotype phasing and for the detection of base modifications from sequencing data [22]. However, its broad use as a mainstream tool in clinical settings is under question since it depends on using high-quality DNA, and in terms of cost, accuracy, throughput, and speed [22]. 

Finally, there is also a statistically significant difference in XLD and XLR disease distribution between X chromosome CGI− and CGI+ genes. XLD disorders are found in CGI+ genes that are not susceptible to methylation, while XLR disorders are found in CGI− genes that are susceptible to methylation. However, these associations did not provide information on the mono-allelic or bi-allelic status of X-linked CGI+ or CGI− genes when this parameter was included in the analysis. Approximately 15% of the tested genes showed bi-allelic expression in accordance with the reported frequency for X-linked genes according to their X-inactivation pattern [23,24]. Therefore, the inactivation of a class of X-linked genes in females leading to mono-allelic expression appears to be controlled by more complex molecular pathways compared to autosomal genes and goes beyond the methylation status of a gene based on the presence of the promoter CGI− loci [5,25,26,27].

Furthermore, the lack of association of XLD or XLR diseases with the mono-allelic or bi-allelic expression of CGI− and CGI+ genes may indicate a balance in the natural selection of XLD or XLR variants between males and females for X chromosome genes. Males, being hemizygous, express both “Dominant” and “Recessive” genetic variants and are directly exposed to natural selection processes [28]. In contrast, females who are heterozygous for a “Dominant” variant (the common phase) have the opportunity to express this variant to some extent, even in the case of a mono-allelic gene. This “Dominant” variant is subject to natural selection due to X-inactivation following a 50:50 ratio for the dominant vs. normal allele, especially since XLD diseases are associated with CGI+ promoters that are not susceptible to methylation, as shown in this study. On the other hand, a female homozygous for a “Recessive” variant may reduce the expression of the recessive variant in the case of bi-allelic genes through passive methylation, controlled by epialleles, to a similar extent to males. This is because XLR diseases are linked to genes with CGI− promoters that are susceptible to methylation. Despite the expectation that the same gene on only one X chromosome in males with the same CGI− promoter would be susceptible to methylation, males have been found to exhibit different levels of methylation compared to females in the same X chromosome regions [27].

## 4. Materials and Methods

### 4.1. Chromosomes’ Dominant and Recessive Diseases in CGI− and CGI+ Genes

Recently, Lee et al. reported a list of CGI− and CGI+ genes in their supplementary Table S2B, using computational and experimental criteria (this article is licensed under CC BY 4.0) [13]. The CGI− and CGI+ genes met the following criteria: (i) CGIs defined by sequence characteristics [i.e., GC content ≥ 50%, length > 200 bp, and CG-observed/CG-expected > 0.6, downloaded from the UCSC database (http://genome.ucsc.edu/)] and (ii) experimentally validated CGIs identified by CxxC-affinity purification, which uses a ZF-CxxC (CxxC) domain to specifically capture DNA containing clusters of non-methylated CpGs, followed by parallel sequencing. Genes with transcription start sites (TSS) surrounded by both consensus CxxC binding regions and UCSC-downloaded CGIs (±500 bp) were defined as CGI+ genes, while genes not associated with both elements from 500 bp upstream of TSS through the gene body were defined as CGI− genes [13]. This categorization resulted in promoter-poor CGI genes (CGI−) prone to methylation and promoter-enriched CGI genes (CGI+) not prone to methylation. Both categories are not related to the intragenic and intergenic CGI sites that are highly variably methylated and exhibit tissue-specific DNA methylation [9,13]. Therefore, this grouping of genes as CGI− and CGI+ corresponds to the classic general model of DNA methylation at promoter-associated CpG dinucleotides for the regulation of their expression [15,29,30]. 

For each autosomal chromosome and the listed CGI− and CGI+ genes, the author searched the total number of OMIM diseases’ phenotypes with autosomal dominant (AD) inheritance and the total number of OMIM diseases’ phenotypes with recessive (AR) inheritance using the OMIM (Online Mendelian Inheritance in Man) database (https://omim.org, accessed on 1 October 2024). Then, Pearson’s Chi-square test was performed for each chromosome to study if there is a difference in the number of counted AD and AR diseases related to CGI− genes vs. the number of the counted AD and AR diseases related to CGI+ genes. The same analysis was conducted for the X chromosome as well. The results are presented in Table 1.

### 4.2. Autosomal Chromosomes’ Dominant and Recessive Diseases in Random or Bi-Allelic Expressed CGI− and CGI+ Genes

The analysis above revealed a significant difference in the distribution of AD and AR diseases among CGI− and CGI+ genes on chromosomes 6 and 17. AD disorders were mainly associated with CGI− genes, which are prone to methylation, while AR disorders were mainly linked to CGI+ genes, which are not susceptible to methylation. In a previous study, the author indicated that chromosomes 6 and 17 fall in the middle of chromosome classification in terms of SNP hotspot regions’ density, which cannot fully explain the imbalance over one variant acting as dominant [1,2]. This suggests that chromosomes within an intermediate level of SNP hotspot frequency, like 6 and 17, may allow for another mechanism, such as methylation, to influence the expression of the so-called “dominant” and “recessive” variants based on whether they involve CGI− or CGI+ genes, respectively. Given that CGI− genes are more susceptible to methylation [9], this could lead to allele-specific expression, a phenomenon frequently observed on chromosomes 6 and 17 [12].

To explore this association further, the author focused on genes known to exhibit random allelic imbalance across all tissue cells, as documented in supplementary Table S3 from Kravitz et al. (this article licensed under CC BY 4.0) [14]. Next, the author identified the overlapping genes between the datasets for CGI− and CGI+ genes [13] as well as for genes showing random allelic imbalance [14] and matched them with AD and AR diseases listed in the OMIM database. In a previous study, the author highlighted the role of low and dense SNP hotspot regions in allelic imbalance, noting that AD diseases tend to occur in low SNP hotspot regions while AR diseases are more common in high SNP hotspot regions [1]. To eliminate this parameter from the current analysis, chromosomes were categorized based on their SNP hotspot density, using the intermediate level of SNP density of chromosomes 6 and 17 as a cutoff point [1,2]. Stratification is a common statistical approach for dealing with confounding effects, which is a major concern in causal studies because it results in a biased estimation of exposure effects [31]. By excluding chromosomes 6 and 17 from this grouping, the aim was to remove bias and focus only on chromosomes with no inherent difference in the distribution of AD and AR diseases among their CGI− and CGI+ genes. This allowed for a focused analysis of CGI− and CGI+ genes displaying random allelic imbalance and mapped on chromosomes with low or high SNP density. The Pearson’s Chi-square test was then conducted to assess differences in the distribution of AD and AR diseases among CGI− and CGI+ genes with random allelic expression in chromosomes with low SNP hotspots (chromosomes: 13, 1, 14, 21, 18, 2, 20, 5, 12, and 15) and high SNP hotspots (chromosomes: 10, 3, 11, 22, 4, 7, 9, 19, 8, and 16). The results are presented in Table 2.

A similar control analysis was performed using the same criteria, this time focusing on genes with bi-allelic expression. The Pearson’s Chi-square test was employed to compare the distribution of AD and AR diseases among CGI− and CGI+ genes with bi-allelic expression in chromosomes with low SNP hotspots (chromosomes: 13, 1, 14, 21, 18, 2, 20, 5, 12, and 15) and high SNP hotspots (chromosomes: 10, 3, 11, 22, 4, 7, 9, 19, 8, and 16). The results are also presented in Table 2.

Furthermore, a Pearson’s Chi-square test was conducted to examine the difference in the distribution of AD and AR diseases between CGI− and CGI+ genes in random allelic and bi-allelic genes on chromosomes 6 and 17. This was undertaken to establish a possible association based on the initially revealed difference (see Table 1). The results of the test are presented in Table 2.

### 4.3. X Chromosome Dominant and Recessive Diseases in CGI− and CGI+ Genes Regarding Their X-Inactivation Process

In relation to the X chromosome, the genes that overlap between the two datasets, namely the CGI− and CGI+ genes [13], which exhibit either monoallelic expression (X-inactivation) or bi-allelic expression (escaping from X-inactivation) [32], were examined. This analysis utilized the combined data of Carrel et al. and Cotton et al., as presented in the supplementary Table S1 by Tukiainen et al. (this article is licensed under CC BY 4.0) [32]. Genes with reported variable expression were excluded from the study. The identified genes were then cross-referenced with X-linked dominant (XLD) and X-linked recessive (XLR) diseases listed in the OMIM database for the X chromosome. A Pearson’s Chi-square test was conducted (Yates’ chi-square was used if any expected frequency was below 1 or if the expected frequency was <5 in more than 20% of cells) to assess differences in the distribution of XLD and XLR diseases: (i) for CGI− genes that either escape or do not escape from X-inactivation, (ii) for CGI+ genes that either escape or do not escape from X-inactivation, (iii) between CGI− and CGI+ genes that do not escape X-inactivation (monoallelic gene expression), and (iv) between CGI− and CGI+ genes that escape X-inactivation (bi-allelic gene expression). The results are detailed in Table 3.

### 4.4. Statistical Analyses

The data used in this study were obtained from published files [13,14,32], which provided the necessary information for the parameters tested. The criteria for classifying all chromosomes and genes were consistent to avoid biases in the analyses. The inheritance pattern of the tested genes (AD, AR, XLD, and XLR) was retrieved from the OMIM database based on records up to 1 October 2024. The statistical analysis was conducted using the IBM SPSS Statistics Version 29.0 (SPSS Inc., Chicago, IL, USA) to perform Pearson’s or Yates’ Chi-square tests and calculate the corresponding odds ratio (OR) values with a 95% confidence interval (CI). The OR values in Table 1, Table 2 and Table 3 indicate the relationship between AD inheritance in autosomal chromosomes or XLD inheritance in the X chromosome and CGI− genes susceptible to methylation in the tested groups. Additionally, in Table 3, the OR values indicating the relationship between XLD inheritance and mono-allelic gene expression in the group of CGI− genes and in the group of CGI+ genes were calculated. A value greater than one for OR suggests a higher likelihood of the tested association. Statistical significance was defined as a difference of *p* ≤ 0.05.

## 5. Conclusions

The previously reported classification of autosomal chromosomes based on SNP hotspot region density from the lowest to the highest is as follows: 13, 1, 14, 21, 18, 2, 20, 5, 12, 15, 17, 6, 10, 3, 11, 22, 4, 7, 9, 19, 8, and 16 [1,2]. This classification can lead to the recognition of: (i) genes susceptible to ASE due to the density of genetic variants [1,2], (ii) promoter CGI− genes in autosomal chromosomes with low SNP hotspots, which may be prone to manifest deleterious variants and related to “Dominant” inheritance due to passive methylation through epialleles, and (iii) genes in chromosomes 6 and 17 showing differences in mode of inheritance between their CGI− and CGI+ genes, with AD diseases mainly found in promoter CGI− genes susceptible to methylation. However, in the case of chromosomes 6 and 17, due to the intermediate level of SNP hotspots, no allelic-specific expression can be predicted. This may explain why the above-mentioned association was revealed for their bi-allelic expressed genes. 

The comprehensive analysis of data for autosomal chromosomes reveals a progression from “chaos” to associating specific patterns of inheritance with CGI−/CGI+ genes on chromosomes with crowded SNPs (10, 3, 11, 22, 4, 7, 9, 19, 8, 16). This leads to associating a specific mode of inheritance in randomly allelic expressed CGI−/CGI+ genes on chromosomes with low SNP density (13, 1, 14, 21, 18, 2, 20, 5, 12, 15), and further to associating a specific mode of inheritance in bi-allelic expressed CGI−/CGI+ genes on chromosomes 6 and 17 with a moderate SNP density. All of these findings appear to align with “The Edge of Chaos” principle in the origin of oscillations in biological systems, explaining complex phenomena [33,34]. Regarding the X chromosome, it is known that the evolutionary dynamics of SNPs on the X chromosome differ from those on autosomes [35]. XLD diseases were primarily found in CGI+ X-linked genes, which are not susceptible to methylation. Therefore, it is not a CGI− loci in the promoter of an X-linked gene related to passive methylation of CpGs through epialleles that could help predict imbalanced expression over the X-allele carrying a disease susceptible variant in heterozygous females. 

Recently, it was reported that the correlation between CpG methylation and gene expression is driven by sequence variants [36]. Specifically, it was reported that a significant fraction of methylated regions is dependent on the presence of heterozygous SNPs in CpG dinucleotides disrupting their methylation potential [6,37]. Therefore, we may need to study inheritance and classify genetic variants for their pathogenicity in a more stochastic way than the terms “Dominant” and “Recessive” and their derivatives like “Codominant” and “Incomplete Dominant”, as applied in Mendelian and non-Mendelian inheritance. This approach could also provide an explanation for the “Reduced Penetrance” and “Variable Expressivity” of some human diseases. Novel sequencing techniques provide accurate detection of CpG methylation in DNA samples and have the benefit of achieving long sequences that facilitate the phasing of variants to parental chromosomes, yielding a haplotype-resolved methylome [36]. All of the above suggests that we need to reconsider the way we study and utilize genetic and epigenetic data for genetic counseling or precision medicine.

## Figures and Tables

**Table 1:** The number of “Dominant” and “Recessive” diseases in CGI− and CGI+ genes across chromosomes.

Chromosome	CGI−		CGI+		*p*-Value [OR (95%CI)]
	Dominant	Recessive	Dominant	Recessive	
**1**	91	118	156	230	0.460 [1.137 (0.809–1.599)]
**2**	54	77	116	179	0.712 [1.082 (0.712–1.646)]
**3**	46	47	129	151	0.570 [1.146 (0.716–1.832)]
**4**	27	41	63	103	0.802 [1.077 (0.604–1.919)]
**5**	31	42	89	98	0.456 [0.813 (0.471–1.403)]
**6**	46	40	66	107	**0.020 [1.864 (1.105–3.146)]**
**7**	36	30	86	97	0.294 [1.354 (0.769–2.381)]
**8**	11	26	58	91	0.302 [0.664 (0.305–1.445)]
**9**	17	22	64	100	0.601 [1.207 (0.596–2.447)]
**10**	20	37	84	97	0.135 [0.624 (0.337–1.157)]
**11**	56	63	111	152	0.376 [1.217 (0.788–1.881)]
**12**	60	58	109	92	0.559 [0.873 (0.554–1.377)]
**13**	5	11	47	42	0.120 [0.406 (0.130–1.265)]
**14**	17	21	63	87	0.761 [1.118 (0.546–2.289)]
**15**	14	21	50	98	0.489 [1.307 (0.613–2.786)]
**16**	17	36	98	117	0.076 [0.564 (0.298–1.065)]
**17**	65	57	102	146	**0.028 [1.632 (1.055–2.525)]**
**18**	7	10	37	38	0.544 [0.719 (0.247–2.089)]
**19**	34	38	98	113	0.909 [1.032 (0.604–1.763)]
**20**	19	12	70	61	0.430 [1.379 (0.619–3.071)]
**21**	2	10	10	37	0.724 [0.740 (0.139–3.936)] 0.964 (Yate’s corrected *p*-value)
**22**	13	17	41	50	0.869 [0.933 (0.406–2.143)]
**X**	15	85	50	139	**0.028 [0.491 (0.259–0.928)]**

CGI−: promoter-poor CpG islands (CGI) genes; CGI+: promoter-enriched CGI genes; OR: odds ratio; 95%CI: 95% confidence intervals.

**Table 2:** The number of “Dominant” and “Recessive” diseases in CGI− and CGI+ genes that exhibit random allelic or bi-allelic gene expression across autosomal chromosomes.

		**Random Allelic Gene Expression**		
		**13 ← 1 ← 14 ← 21 ← 18 ← 2 ← 20 ← 5 ← 12 ← 15 ←**	**← Low SNP density ← 17 — 6 → High SNP density →**	**→ 10 → 3 → 11 → 22 → 4 → 7 → 9 → 19 → 8 → 16**
**CGI−**	**AD**	79	38	61
	**AR**	60	22	62
**CGI+**	**AD**	119	42	119
	**AR**	153	31	164
***p*-value**		**0.012**	0.497	0.160
**OR (95%CI)**		**1.693 (1.121–2.557)**	1.275 (0.633–2.569)	1.356 (0.886–2.074)
		**Bi-Allelic Gene Expression**		
		**13 ← 1 ← 14 ← 21 ← 18 ← 2 ← 20 ← 5 ← 12 ← 15 ←**	**← Low SNP density ← 17 — 6 → High SNP density →**	**→ 10 → 3 → 11 → 22 → 4 → 7 → 9 → 19 → 8 → 16**
**CGI−**	**AD**	142	43	139
	**AR**	171	41	187
**CGI+**	**AD**	435	84	494
	**AR**	555	155	600
***p*-value**		0.658	**0.010**	0.422
**OR (95%CI)**		1.059 (0.821–1.368)	**1.935 (1.169–3.202)**	0.903 (0.703–1.159)

CGI−: promoter-poor CpG islands (CGI) genes; CGI+: promoter-enriched CGI genes; AD: autosomal dominant; AR: autosomal recessive; OR: odds ratio; 95%CI: 95% confidence intervals.

**Table 3:** The number of XLD and XLR diseases present in both CGI− and CGI+ genes across the X chromosome.

		X-Inactivation (Mono-Allelic Gene Expression)	Escaping X-Inactivation (Bi-Allelic Gene Expression)	*p*-Value	OR (95%CI)	*p*-Value (Yate’s Correction)
**CGI−**	**XLD**	7	1	1.00	1.00 (0.104–9.614)	0.564
	**XLR**	42	6			
**CGI+**	**XLD**	30	8	0.048	0.352 (0.125–0.992)	0.081
	**XLR**	96	9			
***p*-value**		0.171	0.158			
**OR (95%CI)**		0.533 (0.217–1.311)	0.188 (0.018–1.911)			
***p*-value** **(Yate’s correction)**		-	0.297			

CGI−: promoter-poor CpG islands (CGI) genes; CGI+: promoter-enriched CGI genes; XLD: X-linked dominant; XLR: X-linked recessive; OR: odds ratio; 95%CI: 95% confidence intervals.

## Data Availability

The data presented in this study are available in this article.

## Supplementary Materials

The following supporting information can be downloaded at: https://www.mdpi.com/article/10.3390/ijms252413377/s1.

## References

[1] Chatzikyriakidou A. (2024). SNPs as Stochastic Oscillators in Inheritance of Human Diseases. Anticancer Res..

[2] Chatzikyriakidou A. (2024). Novel Insights in Genetic Variant Classification. Anticancer Res..

[3] St Pierre C.L., Macias-Velasco J.F., Wayhart J.P., Yin L., Semenkovich C.F., Lawson H.A. (2022). Genetic, epigenetic, and environmental mechanisms govern allele-specific gene expression. Genome Res..

[4] Cleary S., Seoighe C. (2021). Perspectives on Allele-Specific Expression. Annu. Rev. Biomed. Data Sci..

[5] da Rocha S.T., Gendrel A.V. (2019). The influence of DNA methylation on monoallelic expression. Essays Biochem..

[6] Onuchic V., Lurie E., Carrero I., Pawliczek P., Patel R.Y., Rozowsky J., Galeev T., Huang Z., Altshuler R.C., Zhang Z. (2018). Allele-specific epigenome maps reveal sequence-dependent stochastic switching at regulatory loci. Science.

[7] Jeziorska D.M., Murray R.J.S., De Gobbi M., Gaentzsch R., Garrick D., Ayyub H., Chen T., Li E., Telenius J., Lynch M. (2017). DNA methylation of intragenic CpG islands depends on their transcriptional activity during differentiation and disease. Proc. Natl. Acad. Sci. USA.

[8] Kulis M., Queirós A.C., Beekman R., Martín-Subero J.I. (2013). Intragenic DNA methylation in transcriptional regulation, normal differentiation and cancer. Biochim. Biophys. Acta..

[9] Zeng J., Nagrajan H.K., Yi S.V. (2014). Fundamental diversity of human CpG islands at multiple biological levels. Epigenetics.

[10] Ehrlich M., Lacey M. (2013). DNA methylation and differentiation: Silencing, upregulation and modulation of gene expression. Epigenomics.

[11] Robertson K.D. (2005). DNA methylation and human disease. Nat. Rev. Genet..

[12] Roy D., Calaf G., Hei T.K. (2001). Frequent allelic imbalance on chromosome 6 and 17 correlate with radiation-induced neoplastic transformation of human breast epithelial cells. Carcinogenesis.

[13] Lee J.Y., Davis I., Youth E.H.H., Kim J., Churchill G., Godwin J., Korstanje R., Beck S. (2021). Misexpression of genes lacking CpG islands drives degenerative changes during aging. Sci. Adv..

[14] Kravitz S.N., Ferris E., Love M.I., Thomas A., Quinlan A.R., Gregg C. (2023). Random allelic expression in the adult human body. Cell Rep..

[15] Deaton A.M., Bird A. (2011). CpG islands and the regulation of transcription. Genes Dev..

[16] Webster A.K., Phillips P.C. (2024). Heritable epigenetic variation facilitates long-term maintenance of epigenetic and genetic variation. G3 (Bethesda).

[17] Thurman R.E., Rynes E., Humbert R., Vierstra J., Maurano M.T., Haugen E., Sheffield N.C., Stergachis A.B., Wang H., Vernot B. (2012). The accessible chromatin landscape of the human genome. Nature.

[18] Gutierrez-Arcelus M., Lappalainen T., Montgomery S.B., Buil A., Ongen H., Yurovsky A., Bryois J., Giger T., Romano L., Planchon A. (2013). Passive and active DNA methylation and the interplay with genetic variation in gene regulation. Elife.

[19] Hartono S.R., Korf I.F., Chédin F. (2015). GC skew is a conserved property of unmethylated CpG island promoters across vertebrates. Nucleic Acids Res..

[20] Angeloni A., Bogdanovic O. (2021). Sequence determinants, function, and evolution of CpG islands. Biochem. Soc. Trans..

[21] Pértille F., Da Silva V.H., Johansson A.M., Lindström T., Wright D., Coutinho L.L., Jensen P., Guerrero-Bosagna C. (2019). Mutation dynamics of CpG dinucleotides during a recent event of vertebrate diversification. Epigenetics.

[22] Conlin L.K., Aref-Eshghi E., McEldrew D.A., Luo M., Rajagopalan R. (2022). Long-read sequencing for molecular diagnostics in constitutional genetic disorders. Hum. Mutat..

[23] Balaton B.P., Cotton A.M., Brown C.J. (2015). Derivation of consensus inactivation status for X-linked genes from genome-wide studies. Biol. Sex Differ..

[24] Carrel L., Willard H.F. (2005). X-inactivation profile reveals extensive variability in X-linked gene expression in females. Nature.

[25] Mattimoe T., Payer B. (2023). The compleX balancing act of controlling X-chromosome dosage and how it impacts mammalian germline development. Biochem. J..

[26] Peeters S.B., Posynick B.J., Brown C.J. (2023). Out of the Silence: Insights into How Genes Escape X-Chromosome Inactivation. Epigenomes.

[27] Morgan R., Loh E., Singh D., Mendizabal I., Yi S.V. (2024). DNA methylation differences between the female and male X chromosomes in human brain. bioRxiv.

[28] Schaffner S.F. (2004). The X chromosome in population genetics. Nat. Rev. Genet..

[29] Long H.K., King H.W., Patient R.K., Odom D.T., Klose R.J. (2016). Protection of CpG islands from DNA methylation is DNA-encoded and evolutionarily conserved. Nucleic Acids Res..

[30] Saxonov S., Berg P., Brutlag D.L. (2006). A genome-wide analysis of CpG dinucleotides in the human genome distinguishes two distinct classes of promoters. Proc. Natl. Acad. Sci. USA.

[31] Roberts M.R., Ashrafzadeh S., Asgari M.M. (2019). Research Techniques Made Simple: Interpreting Measures of Association in Clinical Research. J. Investig. Dermatol..

[32] Tukiainen T., Villani A.C., Yen A., Rivas M.A., Marshall J.L., Satija R., Aguirre M., Gauthier L., Fleharty M., Kirby A. (2017). Landscape of X chromosome inactivation across human tissues. Nature.

[33] Chua L.O. (2005). Local activity is the origin of complexity. Int. J. Bifurc. Chaos.

[34] Ascoli A., Demirkol A.S., Tetzlaff R., Chua L.O. (2024). Edge of Chaos Theory Sheds Light Into the All-to-None Phenomenon in Neurons—Part I: On the Fundamental Role of the Sodium Ion Channel. IEEE Trans. Circuits Syst. I Regul. Pap..

[35] Miller R.D., Phillips M.S., Jo I., Donaldson M.A., Studebaker J.F., Addleman N., Alfisi S.V., Ankener W.M., Bhatti H.A., Callahan C.E. (2005). SNP Consortium Allele Frequency Project. High-density single-nucleotide polymorphism maps of the human genome. Genomics.

[36] Stefansson O.A., Sigurpalsdottir B.D., Rognvaldsson S., Halldorsson G.H., Juliusson K., Sveinbjornsson G., Gunnarsson B., Beyter D., Jonsson H., Gudjonsson S.A. (2024). The correlation between CpG methylation and gene expression is driven by sequence variants. Nat. Genet..

[37] Shoemaker R., Deng J., Wang W., Zhang K. (2010). Allele-specific methylation is prevalent and is contributed by CpG-SNPs in the human genome. Genome Res..

